# Predictive factors for visual prognosis in neurosyphilis presenting with optic atrophy: a Chinese case series study

**DOI:** 10.3389/fneur.2025.1503956

**Published:** 2025-02-24

**Authors:** Mingjie Zhu, Xin Gu, Pingyu Zhou, Yan Yan

**Affiliations:** ^1^Shanghai Jiao Tong University, School of Medicine, Shanghai, China; ^2^STD Institute, Shanghai Skin Disease Hospital, Tongji University School of Medicine, Shanghai, China; ^3^Eye Institute and Department of Ophthalmology, Eye & ENT Hospital, Fudan University, Shanghai, China

**Keywords:** cerebral spinal fluid, neurosyphilis, optic atrophy, visual acuity, visual field

## Abstract

**Background:**

We aimed to explore the clinical features and predictive factors for visual prognosis of neurosyphilis-associated optic atrophy (NSAOA).

**Methods:**

This retrospective observational study included 17 patients (33 eyes) with NSAOA who received standard anti-ocular syphilis treatment. LogMAR (logarithm of the minimum angle of resolution) best-corrected visual acuity (BCVA), visual field, and optical coherence tomography, were recorded at baseline, short-term (within one month after treatment), and long-term (> 6 months) follow-up. Patients with at least one eye with LogMAR BCVA of ≥1.3 at the last follow-up visit were categorized as the blind group. A change ≥0.2 on the LogMAR BCVA indicated improvement or deterioration.

**Results:**

The mean age was 58.5 years, and 15 patients were males. The mean time between the onset and treatment was 10.1 months. Thirteen patients had Argyll-Robertson pupils. The unblinded group had younger age, shorter disease duration, better baseline visual acuity, higher baseline cerebrospinal fluid (CSF) venereal disease research laboratory (VDRL) titer and CSF total protein counts than the blind group. BCVA of most eyes improved after treatment but experienced deterioration during the follow-up. The deteriorated group of eyes had lower baseline visual field parameters, thinner inferior peripapillary retinal nerve fiber layer (RNFL) thickness. The long-term LogMAR BCVA moderately negatively correlated with CSF VDRL titers before and after treatment.

**Conclusion:**

The diagnosis is often delayed in NSAOA, and the overall visual prognosis is poor. Older age, longer symptom duration, worse baseline vision, thinner RNFL thickness, and lower CSF VDRL titer and total protein counts are significantly associated with worse long-term visual prognosis. The correlation between syphilis serologic test and visual prognosis is poor. It is recommended to reexamine CSF in the follow-up.

## Introduction

Neurosyphilis, a disease that can occur in any stage of syphilis, is caused by the infection of the spirochaete, *Treponema pallidum*, into the central nervous system. Although the reported cases seem to be less than in the pre-antibiotic area due to the widespread use of antibiotics, the prevalence of syphilis and neurosyphilis has experienced a resurge in recent years (1), which is mainly attributed to the risky homosexual behavior between men and the comorbidity of the human immunodeficiency virus (HIV) (2). In China, the incidence of syphilis is second only to viral hepatitis and tuberculosis among infectious diseases (3). And according to the research in Shanghai Skin Disease Hospital, 18.6% of syphilis patients have neurosyphilis (4). Ocular syphilis is defined as clinical symptoms or signs consistent with ocular disease with syphilis of any stage according to the Centers for Disease Control and Prevention (CDC) (5). Ocular syphilis can involve multiple parts of eyeball, especially uveitis is a common manifestation (6). The optic nerve, as a part of the central nerve system, can also be affected in ocular syphilis with patients developing optic neuritis, atrophy, and disc swelling (7). Although ocular syphilis patients with optic nerve involvement can have normal cerebral spinal fluid (CSF) results, some of them can have elevated CSF venereal disease research laboratory (VDRL) results, thus falling into the classification of neurosyphilis. Still, ocular syphilis and neurosyphilis share a standard therapy, which is both treated with intravenous penicillin (7).

Since the first reported case of neurosyphilis patients presenting optic atrophy in 1945 (8), optic atrophy has been described as one of the ocular manifestations of neurosyphilis. According to a 6-year case series study in China, patients with optic atrophy accounted for about 30% of neurosyphilis (9). Optic atrophy can occur at any stage of neurosyphilis but more frequently occurs in late-stage patients with tabes dorsalis (10). Neurosyphilis with optic atrophy commonly manifests as chronic and occult vision loss with a subacute deterioration if not treated promptly. These patients usually exhibit Argyll-Robertson pupil, a characteristic sign of neurosyphilis. The differential diagnoses include glaucoma, optic neuritis, ischemic optic neuropathy (ION), toxic and hereditary optic neuropathy. Although optic atrophy can also be secondary to syphilitic intraocular inflammation, the visual prognosis of uveitis and optic atrophy is particularly different since the visual outcome is often better among uveitis patients (11). To our knowledge, there are few studies reporting neurosyphilis presenting optic atrophy as the isolated manifestation, and the predictive factors for the visual prognosis are unknown. Here, we use the term neurosyphilis-associated optic atrophy (NSAOA), which specifically refers to the optic atrophy caused by neurosyphilis and excludes the secondary optic atrophy from intraocular inflammation or papilledema due to intracranial pressure, and we performed a retrospective study to reveal the clinical features and predictive factors for the visual prognosis of the NSAOA patients.

## Method

### Ethics statement

This retrospective case series study included the patients evaluated and treated at Shanghai Skin Disease Hospital between June 2015 and September 2023 with a diagnosis of neurosyphilis. All these patients were diagnosed with NSAOA and received ophthalmic evaluations at Eye & ENT Hospital. This study was approved by the Shanghai Skin Disease Hospital and Eye & ENT Hospital, Fudan University Ethics Committee.

### Subjects

**Figure 1:**
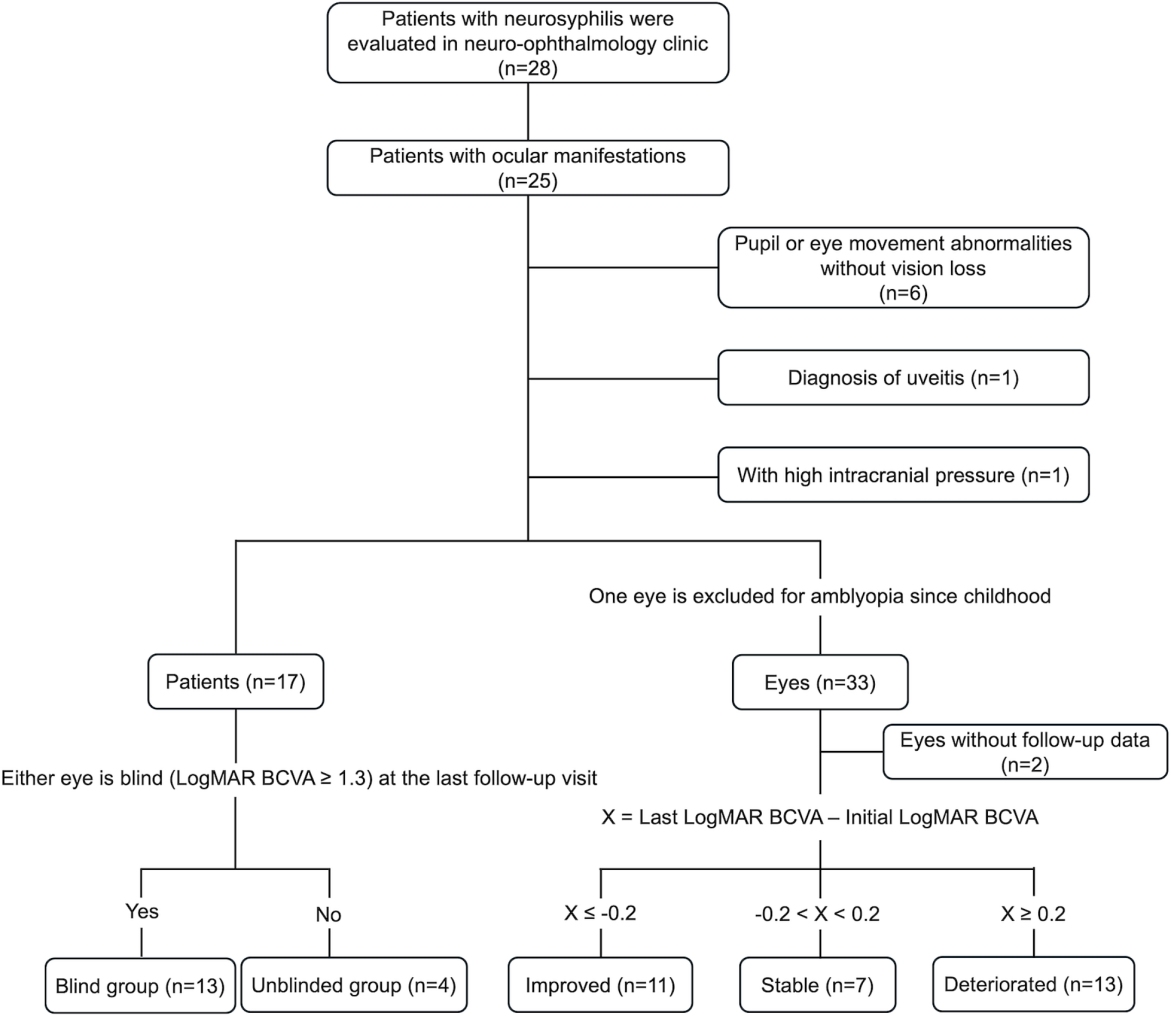
Selection process and grouping procedures of the patients. CSF, cerebral spinal fluid; BCVA, best-corrected visual acuity.

Patients with NSAOA were included if they met the following criteria: (1) Patients had decreased vision or visual field impairment as their chief complaint, (2) Patients were diagnosed with neurosyphilis when they had both positive serum toluidine red unheated serum test (TRUST) and serum treponema pallidum particle agglutination assay (TPPA), and positive CSF VDRL, (3) Patients were diagnosed with optic atrophy based on visual acuity and visual field evaluation, fundus exam, optical coherence tomography (OCT), and magnetic resonance imaging (MRI) results, excluding any other secondary reasons, such as any retinal outer layer abnormalities, retinal or choroidal folds, or retinal edema on OCT, (4) No family history of any optic neuropathy, (5) No history of drug-related optic neuropathy. Exclusion criteria included a previous history of uveitis, potential signs of uveitis (such as cells in the anterior chamber or vitreous, macular edema, retinal lesions, papillitis, or any leakage on fundus fluorescein angiography), optic disc edema on examination or in documentation, or high intracranial pressure on CSF examination. We also excluded other optic neuropathies, including glaucoma, demyelinated or inflammatory optic neuritis, arteritic or non-arteritic ischemic optic neuropathy, papilledema, metabolic optic neuropathy, and hereditary neuropathy. The patient selection process is shown in Figure 1.

Detailed medical records including gender, age, symptom duration, comorbidity, treatment, follow-up time, complete ophthalmic examination (visual acuity, intraocular pressure, eye movement, slit-lamp examination, pupil size, light and near reflex of the pupil, fundus examination), visual field testing, OCT examination, and laboratory tests of serum and CSF samples were collected and analyzed.

### Visual acuity

Visual acuity examination included baseline best corrected visual acuity (BCVA), BCVA measured within one month after treatment, and BCVA at the last follow-up. Each BCVA was transformed into a logarithm of the minimum angle of resolution (LogMAR) value according to the standard logarithmic visual acuity chart. The value of LogMAR for hand movement, light perception, and no light perception was 2.3, 2.8, and 3.0, respectively.

### Visual field testing

Static automated perimetry (Octopus 900 automated visual field analyzer, Haag-Streit AG, Switzerland) were used to measure baseline mean sensitivity (MS) and mean deviation (MD), providing quantitative information regarding the entire visual field. Square-root of the loss variance (sLV) was employed to assess local retinal sensitivity. All visual field tests were conducted under standardized examination conditions. Reliability indices for each test were meticulously recorded to eliminate unreliable data, ensuring the credibility of the results. Poor reliability was deemed present with fixation losses, false positive, or false negative responses of more than 30%.

### OCT examination

In-depth evaluation of retinal structure was achieved through OCT, utilizing the high-resolution OCT instrument from Topcon Corporation. OCT scans yielded multi-layer cross-sectional images of the retina, encompassing detailed information about the ganglion cell layer (GCL) and retinal nerve fiber layer (RNFL). We quantified the average thickness of the GCL and RNFL and further recorded thickness measurements for specific quadrants of the RNFL, including the superior, nasal, inferior, and temporal regions.

### Serum and CSF test for neurosyphilis

All the patients underwent serum tests of TRUST and lumbar puncture for CSF tests, including CSF VDRL titer, CSF white blood cell (WBC) count, CSF total protein, and CSF glucose. Serum and CSF parameters were reevaluated three months and six months after treatment, respectively. Both baseline and follow-up data are recorded and compared.

### Treatments

The treatment of our patients followed the Sexually Transmitted Infections Treatment Guidelines and was one of the following: (i) aqueous crystalline penicillin G, 4 million units intravenously every 4 h for 14 days, (ii) ceftriaxone intravenously, 1 g every 12 h for 14 days if the patient was allergic to penicillin, or (iii) oral doxycycline 200 mg daily for one month if the patient was allergic to penicillin and ceftriaxone (12). Some patients received adjunctive therapy with corticosteroids, most treated with intravenous methylprednisolone followed by oral prednisone.

### Grouping and statistics analysis

Based on the visual outcomes of the patients and eyes, we divided the patients and eyes into different groups for intergroup comparison to determine the factors related to the visual prognosis. According to the WHO definition of blindness, we considered eyes with a LogMAR value of ≥1.3 as blind eyes (13). We defined patients with at least one blind eye at the last follow-up visit as the blind group, and patients with neither of their eyes having LogMAR BCVA ≥1.3 as the unblinded group. LogMAR BCVA of the worse eye was selected in the patient-based data for statistics analysis. The eyes were separated into three groups, which had improved, stable, and deteriorated visual acuity according to the visual acuity changes between the initial and last visits. A change ≥0.2 on the LogMAR value of BCVA indicated improvement or deterioration; otherwise, it was stable.

The statistical analysis was performed with IBM SPSS Statistics 20 software and performed using GraphPad Prism (version 8.2.1). Correlation between factors was calculated using two-tailed Spearman correlation analysis, and the results were recorded as Spearman’s rho (denoted as r_s_). The r_s_ value ranged from −1 to 1, and for the cut-off value, we defined an absolute value of r_s_ > 0.7 as a strong correlation, 0.5 < r_s_ ≤ 0.7 as a moderate correlation, 0.3 < r_s_ ≤ 0.5 as a fair correlation, and rs ≤ 0.3 as a poor correlation (14). Differences between two groups of patients were compared using Mann–Whitney U Test and Chi-square test. Differences among three groups of eyes were compared using one-way analysis of variance (ANOVA) test, and Bonferroni test was used to compare the differences between groups further. *p* value of <0.05 was considered to be statistically significant.

## Results

### Demographic data

Seventeen patients with NSAOA were identified, with a total of 33 eyes included (Supplementary Tables S1, S2). The right eye in Case 1 was excluded because of poor vision since childhood. There were 15 males and two females, with a mean age of 58.5 years. The mean duration between the symptom’s onset and first eye clinical visit was 3.4 months and the mean clinical course before anti-neurosyphilitic treatment was 10.1 months. Nine of 17 patients had bilateral simultaneous visual loss. Thirteen of 17 patients had Argyll-Robertson pupils, and four of 17 patients had positive RAPD. Of the 33 eyes, the mean initial LogMAR BCVA was 1.39. Thirty-one eyes had abnormal visual field testing. The average MS, MD, and sLV were 4.5 dB, 22.4 dB, and 7.9 dB, respectively. For the OCT examination, the average thickness of GCL and RNFL was 54.1 μm and 59.9 μm, and the average thickness for the superior, nasal, inferior, and temporal quadrant of RNFL was 66.6 μm, 56.0 μm, 72.3 μm and 45.0 μm, respectively. One patient (Case 7) was also diagnosed with left third cranial nerve palsy at the initial visit. The third cranial nerve palsy was recovered after treatment, but the vision loss remained.

One patient also had tabes dorsalis. Twelve of 17 patients had comorbidity, including high blood pressure (*n* = 9), diabetes mellitus (*n* = 5), mycoplasma infection (*n* = 1), and systemic lupus erythematosus (*n* = 1). Baseline median serum TRUST titer was 1:64, ranging from 1:8 to 1:256. Baseline median CSF VDRL titer was 1:4, ranging from 1:1 to 1:16. Baseline mean CSF WBC, CSF total protein, and CSF glucose level was 87.9 × 10^6^/L, 766.0 mg/L, and 3.7 mmol/L, respectively. Fifteen patients received aqueous crystalline penicillin G, 4MU intravenously every 4 h for 14 days. One patient (Case 14) received ceftriaxone 1 g every 12 h intravenously for 14 days, and one patient (Case 9) received oral doxycycline 200 mg daily for one month. Four patients received adjunctive therapy with intravenous methylprednisolone followed by oral prednisone. Within one month after treatment, the mean post-treatment LogMAR BCVA was 1.21. The median serum TRUST and CSF VDRL titer were 1:16 and 1:2, respectively. Fifteen patients had decreased titers in both serum TRUST and CSF VDRL. Two patients (Case 7 and Case 11) showed an increased titer of 1:2 to 1:4 and 1:4 to 1:8 in CSF VDRL, despite their decreased serum TRUST titer from 1:64 to 1:16 after treatment. All the patients showed decreased CSF WBC and CSF total protein after treatment. The mean follow-up time was 7.6 months. The mean LogMAR BCVA at the last follow-up visit was 1.56.

### Visual and CSF parameters for prognosis prediction

Intergroup comparison was done on the patient-based and eye-based data according to the group methods, respectively, and the results were shown in Tables 1, 2. Compared to that of the unblinded group, factors showing significant differences in the blind group included older age (*p* = 0.045), longer durations from onset of vision loss to treatment (*p* = 0.032), worse initial LogMAR BCVA (*p* = 0.015), worse post-treatment LogMAR BCVA (*p* = 0.003), worse last LogMAR BCVA (*p* = 0.001) and lower CSF VDRL titer (*p* = 0.003) and CSF total protein (*p* = 0.042). Our results indicated that patients with older age, long symptom duration, worse pre-treatment and post-treatment visual acuity, and lower CSF VDRL titer and total protein tended to have a worse visual prognosis.

**Table 1:** Demographic features of patients with neurosyphilis-associated optic atrophy.

Variables	Blind group^a^ (*n* = 13)	Unblinded group^a^ (*n* = 4)	*p* value
Age (y)	60 (57–65)	56 (54.75–56.25)	0.045*
Sex			0.383
Male (*n*)	12 (92.3%)	3 (75%)	
Female (*n*)	1 (7.7%)	1 (25%)	
Simultaneous binocular involvement			0.475
Yes (*n*)	6 (46.2%)	3 (75%)	
No (*n*)	7 (53.8%)	1 (25%)	
Symptom duration			
Before first visit (m)	3 (2–5)	2 (1–3.25)	0.549
Before treatment (m)	8 (4–12)	2.5 (1.75–3.75)	0.032*
LogMAR BCVA			
Initial	2.3 (1.7–2.8)	0.8 (0.55–1.08)	0.015*
Post-treatment	1.8 (1.22–2.8)	0.34 (0.13–0.62)	0.003*
Last	2.8 (2.18–3)	0.43 (0.13–0.7)	0.001*
Argyll-Robertson pupils			0.267
Yes (*n*)	9 (69.2%)	4 (100%)	
No (*n*)	4 (30.8%)	0 (0%)	
RAPD (+)			0.182
Yes (*n*)	4 (30.8%)	0 (0%)	
No (*n*)	9 (69.2%)	4 (100%)	
Laboratory results			
Serum TRUST titer	1:64 (1:32–1:128)	1:64 (1:64–1:64)	1.000
CSF VDRL titer	1:4 (1:2–1:8)	1:16 (1:14–1:16)	0.003*
CSF WBC (x10^6^/L)	65 (32.75–112.75)	118 (52.75–200.75)	0.350
CSF Total Protein (mg/L)	655.5 (498.63–768.48)	1316.1 (1109.58–1381.35)	0.042*
CSF glucose (mmol/L)	3.5 (2.87–3.92)	3.18 (2.9–5.04)	0.937

The continuous variables of patient-based factors are presented in median (interquartile range). ^a^Patients with at least one blind eye (LogMAR BCVA ≥ 1.3) at the last follow-up visit were categorized as the blind group, and patients with neither of their eyes having LogMAR BCVA ≥ 1.3 were categorized as the unblinded group. ^*^p < 0.05. y, years; *n*, number; m, months; BCVA, best-corrected visual acuity; RAPD, relative afferent pupillary defect; TRUST, serum toluidine red unheated serum test; VDRL, venereal disease research laboratory; WBC, white blood cell.

**Table 2:** LogMAR best-corrected visual acuity, visual field parameters, and optical coherence tomography parameters of the eyes with neurosyphilis-associated optic atrophy.

Variables	Improved^a^ (*n* = 11)	Stable^a^ (*n* = 7)	Deteriorated^a^ (*n* = 13)	*p* value
LogMAR BCVA				
Initial	1.17 ± 0.22	1.56 ± 0.38	1.48 ± 0.25	0.581
Post-treatment	0.66 ± 0.18	1.41 ± 0.41	1.51 ± 0.24	0.050
Last	0.71 ± 0.22	1.59 ± 0.38	2.28 ± 0.21	0.000*
Symptom duration (m)	5.45 ± 1.38	5.29 ± 2.31	12.08 ± 4.55	0.269
Visual field				
MS (dB)	7.17 ± 1.55	4.42 ± 2.24	2.87 ± 0.80	0.071
MD (dB)	19.73 ± 1.56	22.70 ± 2.30	23.99 ± 0.83	0.080
sLV (dB)	6.10 ± 0.63	4.90 ± 1.15	11.12 ± 5.77	0.588
OCT parameters				
Average GCL (μm)	53.60 ± 2.25	52.40 ± 2.54	53.73 ± 1.42	0.911
Average RNFL (μm)	64.00 ± 2.63	60.67 ± 4.25	56.92 ± 2.47	0.195
Superior RNFL (μm)	72.50 ± 3.84	68.83 ± 8.42	61.08 ± 3.29	0.171
Nasal RNFL (μm)	49.90 ± 3.33	61.67 ± 4.93	57.75 ± 4.15	0.174
Inferior RNFL (μm)	87.70 ± 6.18	69.17 ± 7.86	62.42 ± 4.90	0.012*
Temporal RNFL (μm)	45.50 ± 2.43	43.33 ± 2.65	47.00 ± 1.80	0.567

^a^The eyes were separated into improved, stable, and deteriorated groups according to the visual acuity changes between the initial and last visits. A change greater than or equal to 0.2 on the LogMAR value of BCVA indicated improvement or deterioration; otherwise, it was stable. ^*^p < 0.05. BCVA, best-corrected visual acuity; m, months; dB, decibel; MS, mean sensitivity; MD, mean deviation; sLV, short-wavelength automated perimetry; OCT, optical coherence tomography; GCL, ganglion cell layer; RNFL, retinal nerve fiber layer.

Factors showing significant difference between the improved, stable, and deteriorated groups of the eyes included the last LogMAR BCVA (*p* < 0.001) and the inferior thickness of RNFL (*p* = 0.012). Further Bonferroni correction showed the differences of last LogMAR BCVA (*p* < 0.001) and inferior peripapillary RNFL thickness (*p* = 0.011) between the improved and the deteriorated group were significant (not shown in the Table 2). The comparison of the post-treatment LogMAR BCVA (*p* = 0.050), the initial MS (*p* = 0.071) and MD (*p* = 0.080) among three groups had a *p*-value less than 0.1. Taken together, the OCT RNFL thickness results may be important indicators for the visual prognosis.

### The mean visual acuity increased by 2 lines after treatment and then decreased by 3 lines during follow-up

**Figure 2:**
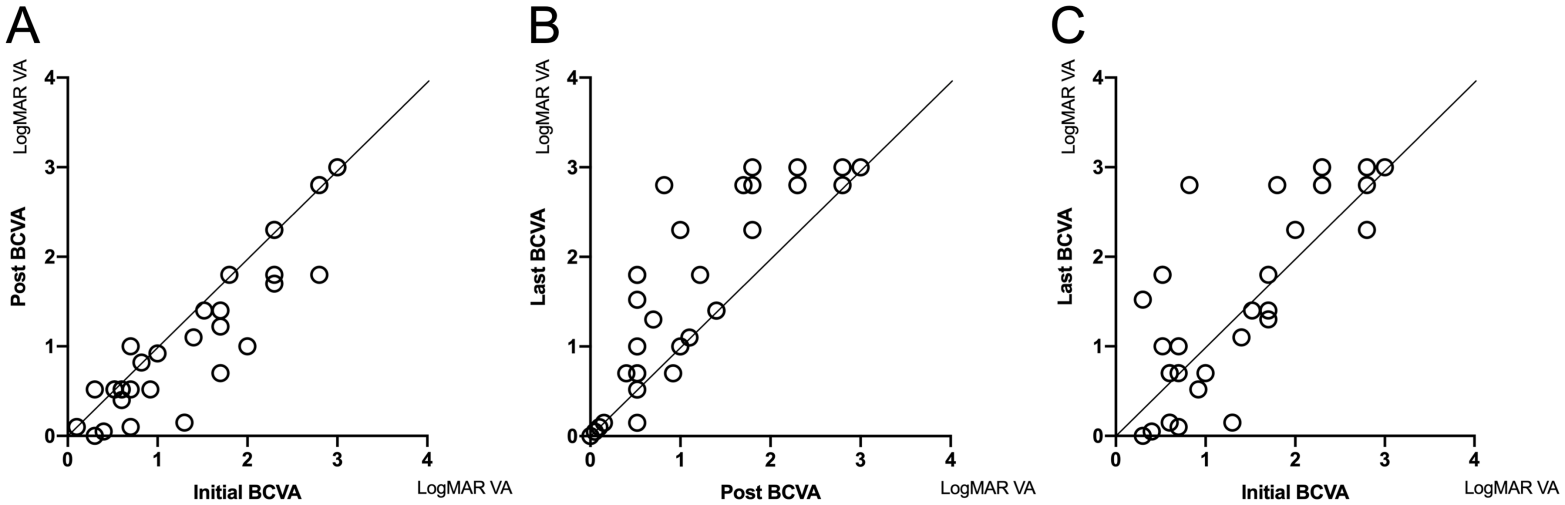
The changes in LogMAR BCVA of the eyes with neurosyphilis-associated optic atrophy among different time points during follow-up. The points below the coordinates’ oblique line (y = x) indicated improvement in LogMAR BCVA. BCVA in most eyes improved after treatment **(A)** but experienced deterioration again during the follow-up **(B)**. If the initial LogMAR BCVA was larger than 2, there was little chance of recovery during long-term follow-up **(C)**. BCVA, best-corrected visual acuity.

The mean initial, post-treatment, and last LogMAR BCVA were 1.39, 1.21, and 1.56, respectively. At the last follow-up visit, 11, 7, and 13 eyes were improved, stable, and deteriorated, respectively (2 eyes lacked follow-up data). Thirteen of 17 patients had at least one eye meeting the criteria of blindness (LogMAR BCVA ≥1.3). Figure 2 shows LogMAR BCVA changes of the eyes at different time points during follow-up. The points below the coordinates’ oblique line (y = x) indicated improvement in LogMAR BCVA. It showed that eyes with NSAOA tended to have temporary improvement shortly after anti-syphilitic treatment but would deteriorate again in the long-term follow-up, which indicated an overall poor visual prognosis. According to Figure 2C, there seemed to be little chance for the eyes with an initial LogMAR BCVA larger than 2, which equaled a visual acuity worse than 20/2000, to recover.

### Correlation analysis showed factors for visual prognosis prediction before and after treatment

**Figure 3:**
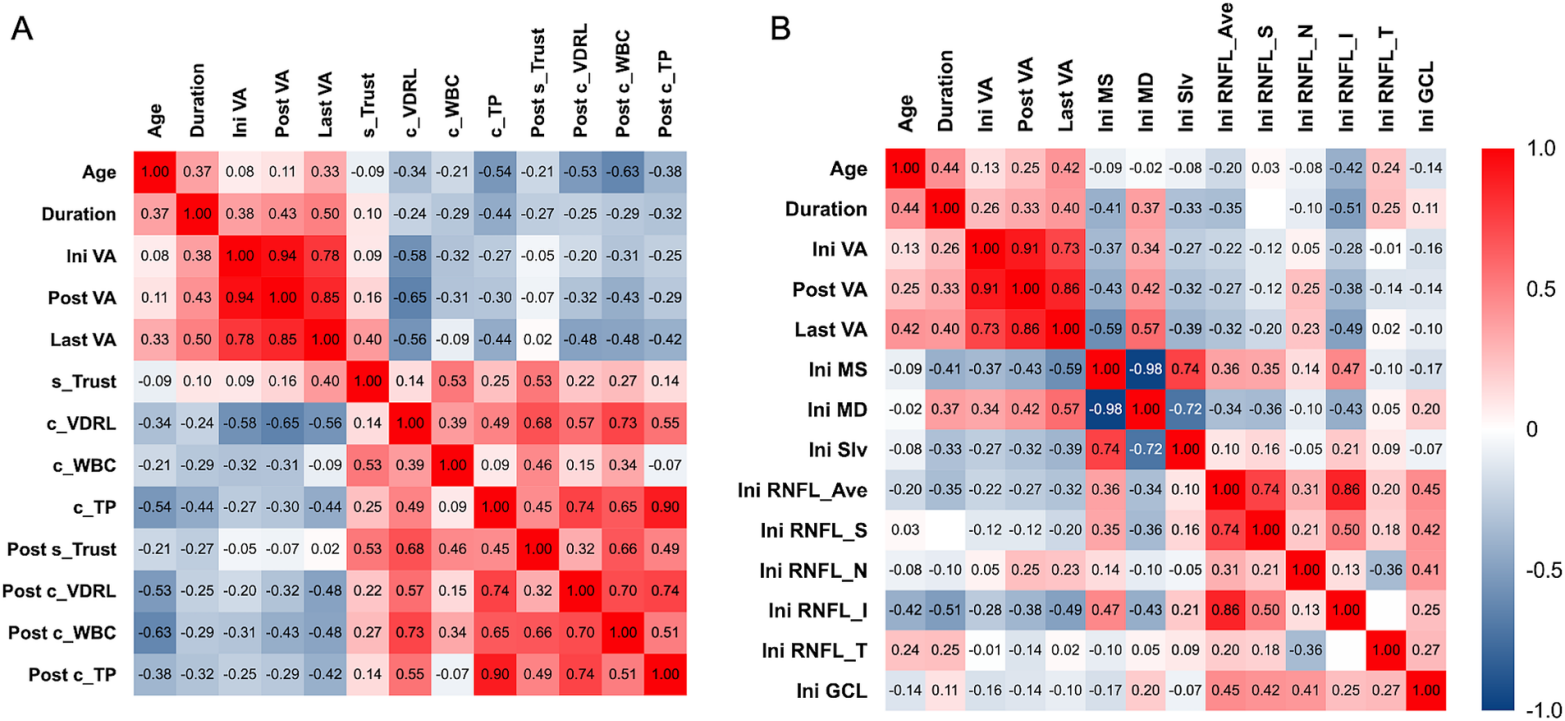
Correlation analysis between LogMAR BCVA of the eyes with optic atrophy associated with neurosyphilis among different time points during follow-up with **(A)** patient-based and **(B)** eye-based factors in our study. **(A)** Baseline CSF VDRL showed moderate correlation with LogMAR BCVA at all time points. Symptom duration, baseline CSF total protein, and post-treatment CSF parameters had fair to moderate correlation with last LogMAR BCVA. **(B)** Baseline VF parameters including MS and MD, and baseline inferior peripapillary RNFL thickness had fair to moderate correlation with last LogMAR BCVA. Ini VA, initial logMAR best-corrected visual acuity; Post VA, post-treatment logMAR best-corrected visual acuity; Last VA, logMAR best-corrected visual acuity at the last follow up; s_TRUST, initial serum toluidine red unheated serum test titer; c_VDRL, initial cerebral spinal fluid titer venereal disease research laboratory titer; c_WBC, initial cerebral spinal fluid white blood cell count; c_TP, initial cerebral spinal fluid total protein; Post s_TRUST, post-treatment serum toluidine red unheated serum test titer; Post c_VDRL., post-treatment cerebral spinal fluid titer venereal disease research laboratory titer; Post c_WBC, post-treatment cerebral spinal fluid white blood cell count; Post c_TP, post-treatment cerebral spinal fluid total protein; Ini MS, initial mean sensitivity; Ini MD, initial mean deviation; Ini sLV, initial short-wavelength automated perimetry; Ini RNFL_Ave, initial average thickness of retinal nerve fiber layer; Ini RNFL_S, initial thickness of the superior quadrant of retinal nerve fiber layer; Ini RNFL_N, initial thickness of the nasal quadrant of retinal nerve fiber layer; Ini RNFL_I, initial thickness of the inferior quadrant of retinal nerve fiber layer; Ini RNFL_T, initial thickness of the temporal quadrant of retinal nerve fiber layer; Ini GCL, initial thickness of ganglion cell layer.

Correlation analyses between parameters of vision and syphilis antibody in sera and CSF at different time points and the other factors in our study are shown in Figure 3. The LogMAR BCVA after treatment (r_s_ = 0.94) and the last LogMAR BCVA (r_s_ = 0.78) showed a strong positive correlation with the initial LogMAR BCVA, which indicated that the long-term visual prognosis strongly positively correlated to the initial visual acuity.

Among the baseline factors, we found that CSF VDRL titer before treatment had a moderately negative correlation with LogMAR BCVA at any time points, which meant lower initial CSF VDRL titer correlated with worse vision. Other factors, including symptoms duration, initial serum Trust titer, CSF total protein, MS, MD, and inferior peripapillary RNFL thickness had fair to moderate correlation with the LogMAR BCVA at the last follow-up (Figures 3A,B). Among the post-treatment factors, CSF parameters had a fair correlation with the last LogMAR BCVA. In contrast, serum TRUST titer did not correlate with LogMAR BCVA, indicating that we should focus more on the CSF test results. Interestingly, patients with better visual acuity tended to have higher CSF VDRL titer, WBC, and total protein counts.

## Discussion

Optic atrophy is one of the most severe manifestations of ocular syphilis, and it often occurs in the late stage of syphilis infection, where the *Treponema pallidum* invades the central nerve system and leads to neurosyphilis diagnosis. Our study showed that optic atrophy can present without other common late stage neurosyphilis manifestations like tabes dorsalis and general paresis. This is the largest case series so far since these patients have previously only been noted in individual case reports or small case series (9, 11, 15, 16). Gu et al. reported eyes with optic nerve involvement were more likely to lead to blindness than those with uveitis (11). However, subgroup analysis was lacking. Sun et al. reported the eyes with optic atrophy in ocular syphilis showed poor visual recovery after treatment (9). Unfortunately, they did not indicate whether these eyes with optic atrophy were from patients with neurosyphilis. This study systematically explored the visual prognostic factors of neurosyphilis with optic atrophy for the first time, emphasizing the potential role of CSF VDRL titer, OCT and visual field. In our series, most cases were middle-aged or older males who experienced insidious vision loss or visual field defect over weeks to months before they were found to have neurosyphilis. Ocular manifestation can be the only presenting feature in the secondary and tertiary stages of syphilis without significant systemic symptoms like mucocutaneous lesions or lymphadenopathy, which can be easily overlooked during physical examination (17). Thus, we demonstrated the importance of considering neurosyphilis, especially in men over 50 with unexplained insidious vision loss and optic atrophy.

The insidious vision loss of NSAOA can easily lead to misdiagnosis. Differential diagnosis should include glaucoma, optic neuritis, ischemic optic neuropathy, toxic and hereditary optic neuropathy. Medical history should be taken in detail, including the risky sexual behavior and family history. The presence of Argyll-Robertson pupil is important for differential diagnosis since it is especially associated with neurosyphilis and occurs more often in the late stage (17). Over 70% of our patients had Argyll-Robertson pupil, indicating that it is a red flag of NSAOA. One patient showed the third cranial nerve palsy after the onset of vision loss. Although ophthalmoplegia recovered after treatment, the vision did not improve and even deteriorated during follow-up. The palsy of the ocular motor cranial nerve, including the third, fourth, and sixth cranial nerves, is one of the potential ocular manifestations of neurosyphilis (18). Previous studies have shown that ophthalmoplegia could be reversed after in-time penicillin treatment. Still, a larger sample size and longer follow-up period are required for further research for these patients.

Previous studies showed that the visual prognosis in neurosyphilis-associated optic atrophy was usually poor, and the eyes with optic atrophy showed more inadequate response to the treatment of penicillin than the eyes with active intraocular inflammation like posterior uveitis, panuveitis, and papillitis (11, 19). In our study, although the eyes showed temporary improvement after treatment, they deteriorated again quickly during follow-up. Those eyes with an initial BCVA worse than 20/2000 had little chance to improve during long-term follow-up. About 70% of our patients eventually had at least one eye that met the blindness criteria of WHO. Still, some eyes that were treated early showed persistent improvement after treatment. We found the eventually improved eyes had a thicker baseline inferior peripapillary RNFL thickness than the other eyes. It indicated the progressive damage of the *Treponema pallidum* directly or indirectly done to the retinal nerve fibers, thus causing a thinner RNFL and a worse prognosis. Also, the initial visual field MS and MD of the eye before treatment also showed significant differences among the improved, stable, and deteriorated groups of eyes. However, they did not meet the statistical significance, probably because of the limited sample size. Together, these results emphasized the importance of the visual field and OCT examinations in NSAOA for predicting prognosis.

The laboratory examination showed that all patients had an initial serum TRUST titer ≥1:8 with a median of 1:64, and an initial CSF VDRL titer≥1:1 with a median of 1:4, consistent with previous studies in ocular syphilis (9). The proportion of abnormal CSF in optic atrophic patients is higher than that in ocular syphilis patients, indicating a longer and more progressive clinical course of optic atrophy in neurosyphilis (9). According to previous studies, syphilis patients with a higher baseline serum RPR titer had a better response to the treatment (20, 21). Similarly, we found the median CSF VDRL titer was four times higher in the unblinded group (1:16) compared to the blind group (1:4). The median duration from onset of vision loss to treatment in the unblinded group was 5.5 months shorter than the blind group. The patient with a better long-term visual acuity tended to have a higher post-treatment CSF VDRL titer, CSF WBC, and total protein counts. The invasion of *Treponema pallidum* into the central nerve system could initiate the perivascular inflammatory response. As the course goes on, large myelinated nerves could be invaded, resulting in subsequent neuronal degeneration (22). In this way, we hypothesized that the higher baseline CSF parameters in the unblinded group were caused by a more activated inflammatory response in the neurosyphilis patients with a shorter clinical course so that this group of patients had a better response to the earlier intervention, thus having a better visual prognosis. Still, due to the poor correlation between serum TRUST after treatment and long-term visual acuity, we suggested that CSF test was essential for post-treatment surveillance.

The pathological mechanism of how neurosyphilis causes the optic atrophy remains unknown. We hypothesized that the optic neuropathy may be partly caused by ischemia of the optic nerve since obliterative endarteritis has been found in syphilis. Tieger et al. described a case of OCT angiography-proven ischemic injury at the optic nerve head in a patient with *Borrelia burgdorferi* spirochete-associated papillitis (23). Notably, when fundus examination was not able to be obtained at the very early stage (the initial week or two after the vision loss occurred), the optic disc edema may have been missed. Previous studies suggested that the invasion of the large myelinated nerve fibers by *Treponema pallidum* resulted in the degeneration of nerves, which could also be the case in the NSAOA (22). Inflammation can also play a part in the disease course since previous case reports recorded disc edema at the initial stage (24). The underlying mechanism of the NSAOA still requires further investigation to verify.

Our study had several limitations. It was a retrospective study, so the clinical data at the very early stage of the disease was deficient, which may cause the overlook of the initial ophthalmic changes. Moreover, the arrest of optic atrophy occurred from 2 to 15 years after treatment, while the CSF parameters went down and finally became normal after the arrest of optic atrophy (25). So we need a longer follow-up period to evaluate the long-term outcome. The 10 patients with missing CSF tests included 3 patients in the unblinded group and 7 patients in the blind group. Therefore, we did not analyze the comparison of changes in CSF after treatment between the two groups but analyzed the correlation between CSF and vision after treatment. If we could further follow up the changes in CSF, we would have a better understanding of the CSF status of these patients with poor prognosis and whether it is related to poor visual acuity recovery. Due to the research objective, we excluded patients with negative CSF VDRL results. However, for all patients with neurosyphilis, attention should be paid to whether there is optic atrophy. Conversely, for patients with optic atrophy, neurosyphilis should be ruled out. Even if the CSF VDRL is negative, the examination of the composition of CSF and syphilis serological tests are also of great importance.

## Conclusion

The insidious vision loss and Argyll-Robertson pupils are the major manifestations of NSAOA. When diagnosis is delayed, the overall visual prognosis was poor in most patients despite the temporary improvement after treatment. Factors including older age, longer symptom duration, worse baseline visual acuity and VF, thinner retinal nerve fiber layer thickness, and lower CSF VDRL titer, WBC, and total protein counts are associated with worse long-term visual prognosis. The correlation between syphilis serologic test and visual prognosis is poor. It is recommended to reexamine CSF in the follow-up.

## Data Availability

The original contributions presented in the study are included in the article/Supplementary material, further inquiries can be directed to the corresponding authors.
