# Supplementary material for: Predictive factors for visual prognosis in neurosyphilis presenting with optic atrophy: a Chinese case series study
Source: Front Neurol. 2025 Feb 24;16:1503956. doi: 10.3389/fneur.2025.1503956 (PMC11891054; doi:10.3389/fneur.2025.1503956)
Supplement: Supplementary file 1 [file Table_1.docx]

| **Table S1** Patient-based data in Neurosyphilis associated optic atrophy. | | | | | | | | | | | | | | | | | | | | | |
| --- | --- | --- | --- | --- | --- | --- | --- | --- | --- | --- | --- | --- | --- | --- | --- | --- | --- | --- | --- | --- | --- |
| Patient number | Gender (M=male, F=female) | Age (y) | Symptom duration before first visit (m) | Symptom duration before treatment (m) | AR pupil  (Y=yes, N=no) | RAPD+  (Y=yes, N=no) | Serum TRUST titer | Post-treatment serum TRUST titer | CSF VDRL titer | Post-treatment CSF VDRL titer | CSF WBC count (10^6/L) | Post-treatment CSF WBC count (*10^6/L) | CSF total protein (mg/L) | Post-treatment CSF total protein (mg/L) | CSF glucose (mmol/L) | Post-treatment CSF glucose (mmol/L) | Hypertension  (Y=yes, N=no) | Diabetes mellitus (Y=yes, N=no) | Therapy ^a^ | Use of glucocorticoid (Y=yes, N=no) | Follow-up time (m) |
| 1 | M | 57 | 12 | 12 | N | N | 1:64 | 1:16 | 1:8 | 1:4 | 130 | 13 | 690.4 | 449.7 | 3.23 | 3.81 | Y | N | 1 | N | 6 |
| 2 | M | 60 | 3 | 3 | Y | N | 1:32 | NA | 1:4 | NA | 41 | NA | 904.9 | NA | 3.68 | NA | Y | N | 1 | N | 6 |
| 3 | M | 60 | 3 | 18 | N | Y | 1:64 | 1:32 | 1:8 | 1:2 | NA | 8 | NA | 1096 | NA | 4 | N | Y | 1 | N | 7 |
| 4 | F | 57 | 8 | 8 | Y | Y | 1:64 | 1:64 | 1:4 | NA | 190 | NA | 421 | NA | 4 | NA | Y | Y | 1 | Y | 10 |
| 5 | M | 65 | 1 | 6 | Y | N | 1:128 | 1:16 | 1:4 | 0 | 286 | 4 | 176 | 231 | 3.5 | 2.9 | N | N | 1 | Y | 6 |
| 6 | M | 56 | 1 | 2 | Y | N | 1:64 | NA | 1:16 | NA | 263 | NA | 1357.2 | NA | 6.9 | NA | N | Y | 1 | N | 6 |
| 7 | M | 53 | 0.25 | 2 | Y | N | 1:64 | 1:16 | 1:2 | 1:4 | 80 | 8 | 678 | 642 | 4.6 | 4 | N | N | 1 | N | 6 |
| 8 | M | 66 | 0.25 | 4 | Y | Y | 1:128 | 1:64 | 1:8 | NA | 96 | NA | 633 | NA | 2.7 | NA | N | N | 1 | N | 15 |
| 9 | M | 68 | 3 | 60 | Y | Y | 1:128 | NA | 1:4 | NA | 50 | NA | 723 | NA | NA | NA | N | N | 2 | N | 6 |
| 10 | M | 56 | 1 | 3 | Y | N | 1:64 | NA | 1:16 | NA | 43 | NA | 1453.8 | NA | 3.18 | NA | Y | N | 1 | N | 6 |
| 11 | M | 61 | 3 | 4 | Y | N | 1:64 | 1:16 | 1:4 | 1:8 | 107 | 11 | 922.2 | 701.1 | 2.55 | 3.17 | Y | N | 1 | N | 9 |
| 12 | M | 63 | 2 | 9 | N | N | 1:16 | NA | 1:1 | 0 | 0 | 0 | 562 | 411 | 4.2 | 4.2 | Y | Y | 1 | N | 12 |
| 13 | M | 66 | 6 | 5 | Y | N | 1:8 | NA | 1:1 | NA | 8 | NA | 370 | NA | 3.5 | NA | Y | N | 1 | N | 6 |
| 14 | F | 57 | 3 | 6 | Y | N | 1:64 | NA | 1:8 | NA | 56 | NA | 613.3 | NA | 2.62 | NA | Y | N | 3 | N | 6 |
| 15 | M | 40 | 3 | 10 | N | N | 1:256 | NA | 1:2 | NA | 48 | NA | 951 | NA | NA | NA | N | N | 1 | N | NA |
| 16 | M | 51 | 4 | 1 | Y | N | 1:64 | 1:32 | 1:16 | 1:1 | 180 | 24 | 1275 | 525 | NA | 2.8 | N | Y | 1 | Y | 9 |
| 17 | M | 58 | 5 | 18 | Y | N | 1:16 | NA | 1:8 | NA | 6 | NA | 524.5 | NA | 2.75 | NA | N | N | 1 | N | 6 |

y, years; m, months; AR, Argyll-Robertson; NA, not available; RAPD, relative afferent papillary defect; TRUST, toluidine red unheated serum test; CSF, cerebrospinal fluid; VDRL, venereal disease research laboratory; WBC, white blood cell; h, hours; d, days.

a 1=penicillin 4 millionU IV q4h X 14d; 2=Doxycycline PO; 3=Ceftriaxone sodium 1g IV q12h X 14d
