# Supplementary material for: Predictive factors for visual prognosis in neurosyphilis presenting with optic atrophy: a Chinese case series study
Source: Front Neurol. 2025 Feb 24;16:1503956. doi: 10.3389/fneur.2025.1503956 (PMC11891054; doi:10.3389/fneur.2025.1503956)
Supplement: Supplementary file 2 [file Table_2.docx]

| **Table S2.** Eye-based data in neurosyphilis-associated optic atrophy. | | | | | | | | | | | | | | | |
| --- | --- | --- | --- | --- | --- | --- | --- | --- | --- | --- | --- | --- | --- | --- | --- |
| Patient number | Eye Number | Eye laterality (R=right, L=left) | Symptom duration before treatment (m) | Initial logMAR BCVA | Post-treatment logMAR BCVA | Last logMAR BCVA | MS (dB) | MD (dB) | sLV (dB) | Thickness of GCL (μm) | Thickness of average RNFL (μm) | Thickness of superior quadrant RNFL (μm) | Thickness of nasal quadrant RNFL (μm) | Thickness of inferior quadrant RNFL (μm) | Thickness of temporal quadrant RNFL (μm) |
| 1 | 1 | L | 12 | 0.52 | 0.52 | 1.8 | 0.9 | 26.8 | 3.3 | NA | NA | NA | NA | NA | NA |
| 2 | 2 | R | 3 | 1.52 | 1.4 | 1.4 | 13.7 | 13.1 | 5.6 | 50 | 78 | 96 | 81 | 104 | 32 |
| 2 | 3 | L | 3 | 1.7 | 1.4 | 1.4 | 13.8 | 13 | 6.6 | 53 | 68 | 87 | 63 | 94 | 29 |
| 3 | 4 | R | 7 | 0.52 | 0.52 | 1 | 6.2 | 20.8 | 7.7 | 58 | 62 | 77 | 61 | 61 | 50 |
| 3 | 5 | L | 18 | 2.3 | 2.3 | 3 | 0 | 27 | 2.2 | 58 | 63 | 76 | 55 | 64 | 56 |
| 4 | 6 | R | 8 | 2.8 | 2.8 | 2.8 | NA | NA | NA | 47 | 48 | 51 | 44 | 45 | 51 |
| 4 | 7 | L | 8 | 1.7 | 0.7 | 1.3 | NA | NA | NA | 52 | 50 | 54 | 48 | 51 | 46 |
| 5 | 8 | R | 6 | 0.82 | 0.82 | 2.8 | 6.4 | 20.3 | 6.7 | 57 | 63 | 62 | 55 | 82 | 55 |
| 5 | 9 | L | 6 | 0.92 | 0.52 | 0.52 | 14.7 | 12.1 | 6.9 | 66 | 74 | 75 | 71 | 98 | 51 |
| 6 | 10 | R | 2 | 0.7 | 0.1 | 0.1 | 6.5 | 20.5 | 7.4 | 52 | 63 | 75 | 38 | 92 | 47 |
| 6 | 11 | L | 2 | 1.3 | 0.15 | 0.15 | 9.6 | 17.4 | 7.8 | 57 | 79 | 91 | 52 | 119 | 54 |
| 7 | 12 | R | 2 | 1.8 | 1.8 | 2.8 | 1.6 | 26.4 | 4.9 | NA | 74 | 56 | 99 | 106 | 36 |
| 7 | 13 | L | 2 | 3 | 3 | 3 | 0 | 27.9 | 2 | NA | 57 | 60 | 64 | 62 | 42 |
| 8 | 14 | R | 4 | 2.3 | 1.8 | 3 | 1.3 | 25.2 | 4.3 | 50 | 53 | 63 | 46 | 51 | 52 |
| 8 | 15 | L | NA | 0.52 | 0.52 | 1.8 | 4.2 | 22.3 | 7 | 50 | 47 | 51 | 47 | 47 | 42 |
| 9 | 16 | R | 12 | 0.3 | 0.52 | 1.52 | 1.8 | 24.6 | 4 | 57 | 58 | 68 | 64 | 57 | 44 |
| 9 | 17 | L | 60 | 2.8 | 2.8 | 3 | 0 | 26.4 | 2.2 | 47 | 55 | 60 | 53 | 58 | 48 |
| 10 | 18 | R | 3 | 0.4 | 0.05 | 0.05 | 2.5 | 24.5 | 4.4 | 57 | 63 | 55 | 40 | 107 | 50 |
| 10 | 19 | L | 3 | 0.3 | 0 | 0 | 1.8 | 25.2 | 3.7 | 57 | 65 | 75 | 41 | 90 | 52 |
| 11 | 20 | R | 4 | 1.4 | 1.1 | 1.1 | 5.9 | 20.8 | 7.4 | 57 | 62 | 76 | 52 | 75 | 45 |
| 11 | 21 | L | 4 | 1.7 | 1.22 | 1.8 | 0.4 | 26.3 | 3.1 | 62 | 67 | 94 | 58 | 68 | 48 |
| 12 | 22 | R | 7 | 2.3 | 2.3 | 2.8 | 0.6 | 26.4 | 14.6 | 60 | 57 | 64 | 54 | 62 | 49 |
| 12 | 23 | L | 9 | 2.3 | 1.7 | 2.8 | 7.1 | 19.9 | 79.5 | 52 | 59 | 65 | 57 | 66 | 49 |
| 13 | 24 | R | 5 | 2 | 1 | 2.3 | 0 | 26.5 | 2.2 | 47 | 41 | 34 | 43 | 50 | 38 |
| 13 | 25 | L | 3 | 0.7 | 1 | 1 | 7.2 | 19.3 | 5.9 | 55 | 51 | 57 | 59 | 45 | 45 |
| 14 | 26 | R | 5 | 0.6 | 0.52 | 0.15 | 9.3 | 17.6 | 7.8 | 41 | 60 | 74 | 51 | 68 | 45 |
| 14 | 27 | L | 6 | 1 | 0.92 | 0.7 | 7.6 | 19.3 | 6.9 | 44 | 56 | 63 | 43 | 83 | 36 |
| 15 | 28 | R | NA | 0.1 | 0.1 | NA | 3.4 | 24.3 | 5.3 | 67 | 59 | 66 | 59 | 70 | 39 |
| 15 | 29 | L | 10 | 3 | 3 | NA | 1.8 | 25.9 | 4.4 | 59 | 52 | 61 | 59 | 58 | 32 |
| 16 | 30 | R | 1 | 0.6 | 0.4 | 0.7 | 8.1 | 19.1 | 9.1 | 51 | 57 | 60 | 58 | 67 | 44 |
| 16 | 31 | L | 1 | 0.6 | 0.52 | 0.7 | 3.6 | 23.6 | 7 | 52 | 57 | 52 | 65 | 69 | 43 |
| 17 | 32 | R | 18 | 0.7 | 0.52 | 0.7 | 0.7 | 26.2 | 2.6 | NA | NA | NA | NA | NA | NA |
| 17 | 33 | L | 18 | 2.8 | 1.8 | 2.3 | 0 | 26.9 | 2.1 | NA | NA | NA | NA | NA | NA |
| m, months; BCVA, best-corrected visual acuity; MS, mean sensitivity; MD, mean deviation; NA, not available; sLV, square-root of the loss variance; GCL, ganglion cell layer; RNFL, retinal nerve fiber layer. | | | | | | | | | | | | | | | |
